# Mitochondrial fatty acid oxidation regulates monocytic type I interferon signaling via histone acetylation

**DOI:** 10.1126/sciadv.adq9301

**Published:** 2025-01-22

**Authors:** Jing Wu, Komudi Singh, Vivian Shing, Anand Gupta, Brett C. Arenberg, Rebecca D. Huffstutler, Duck-Yeon Lee, Michael N. Sack

**Affiliations:** ^1^ Laboratory of Mitochondrial Biology and Metabolism, National Heart, Lung, and Blood Institute, National Institutes of Health, Bethesda, MD, USA.; ^2^ Cardiovascular Branch, National Heart, Lung, and Blood Institute, National Institutes of Health, Bethesda, MD, USA.; ^3^ Biochemistry Core, National Heart, Lung, and Blood Institute, National Institutes of Health, Bethesda, MD, USA.

## Abstract

Although lipid-derived acetyl–coenzyme A (CoA) is a major carbon source for histone acetylation, the contribution of fatty acid β-oxidation (FAO) to this process remains poorly characterized. To investigate this, we generated mitochondrial acetyl-CoA acetyltransferase 1 (ACAT1, distal FAO enzyme) knockout macrophages. ^13^C-carbon tracing confirmed reduced FA-derived carbon incorporation into histone H3, and RNA sequencing identified diminished interferon-stimulated gene expression in the absence of ACAT1. Chromatin accessibility at the *Stat1* locus was diminished in ACAT1^−/−^ cells. Chromatin immunoprecipitation analysis demonstrated reduced acetyl-H3 binding to *Stat1* promoter/enhancer regions, and increasing histone acetylation rescued *Stat1* expression. Interferon-β release was blunted in ACAT1^−/−^ and recovered by ACAT1 reconstitution. Furthermore, ACAT1-dependent histone acetylation required an intact acetylcarnitine shuttle. Last, obese subjects’ monocytes exhibited increased ACAT1 and histone acetylation levels. Thus, our study identifies an intriguing link between FAO-mediated epigenetic control of type I interferon signaling and uncovers a potential mechanistic nexus between obesity and type I interferon signaling.

## Teaser

Mitochondrial fatty acid oxidation is essential for the epigenetic control of type I interferon signaling.

## INTRODUCTION

Immune cells exhibit diverse metabolic demands contingent upon their proliferative capacity, polarization, and activation status. It is well established that cellular metabolism is highly integrated with immune cell fate and function. The interaction between metabolism and immune function is defined as immunometabolism and encompasses (i) energy substrate utilization (fate-dependent metabolic remodeling) (*1*–*3*), (ii) the role of metabolic intermediates as signaling molecules (*4*–*6*), and (iii) the indirect sequelae of metabolism on intracellular organelle function and fidelity resulting in subsequent retrograde signaling (*7*–*9*). Emerging evidence implicates pentose phosphate pathway and nucleotide synthesis intermediates (*10*), amino acids, long-chain saturated fatty acids, and amino acid and fatty acid conjugates (*11*) in conferring immunoregulation. In addition, nutrient-sensing organelles are similarly implicated in immune regulation through retrograde signaling from mitochondria (*8*, *12*, *13*), autophagosomes (*10*, *14*, *15*), and the endosome-lysosome system (*16*, *17*). Last, immune cells’ sensing of circulating metabolites (*11*, *18*, *19*) and intracellular nutrient availability (*20*, *21*) integrate metabolic pathways with signaling to regulate immune responses.

Recent research has highlighted the emerging role of acetyl–coenzyme A (CoA) as a crucial metabolic immunoregulator mediator. Acetyl-CoA functions as a substrate in balancing fat oxidation and lipid synthesis, thereby modulating immune responsiveness (*22*). It also serves as a substrate for histone acetylation, which leads to chromatin relaxation and activation of immunoregulatory genes (*23*). Numerous metabolites catabolized in mitochondria generate acetyl-CoA, with glucose being a canonical example whereby nutrient-derived carbon integrates into the epigenome through histone acetylation (*24*). Recently, acetyl-CoA derived from lipid oxidation was identified as a major contributor to the global acetyl-CoA pool, which is reflected by increased levels of acetate, citrate, and histone acetylation (*25*). However, direct evidence specifying the enzymes and metabolic intermediates that bridge lipids to acetyl-CoA, their transport mechanisms for histone acetylation, and their specific roles in immunoregulation remains lacking.

To investigate the composite roles of various components of lipid oxidation, we focused on exploring the potential immunoregulatory role of the mitochondrial thiolase acetyl-CoA acetyltransferase 1 (ACAT1) in myeloid cells. As its name implicates, ACAT1 is enriched in mitochondria and functions as a mitochondrial acetyltransferase enzyme (*26*). It is a key distal enzyme in the catabolism of isoleucine (*27*), fatty acids (*28*), and ketone bodies (*29*) to generate acyl-CoA moieties and CoA. We hypothesized that studying this enzyme may provide additional insight into how mitochondrial metabolism and signaling regulate immune cell function.

In this study, we found that type I interferon (T1IFN) signaling was substantially diminished in myeloid cells following the genetic depletion of ACAT1. This effect was due to the reduction in fatty acid–mediated histone acetylation, resulting in attenuation in transcript and protein levels of a canonical T1IFN regulatory protein signal transducer and activator of transcription 1 (STAT1). We confirmed that this modulation of histone acetylation by fatty acid catabolism is dependent on the carnitine/acylcarnitine translocase (CACT). Moreover, in human subjects with obesity, circulating monocytes exhibited higher levels of ACAT1 compared to lean control subjects. This increase was associated with elevated histone acetylation and higher transcript levels of canonical T1IFN pathway genes. These findings collectively indicate that ACAT1-mediated fatty acid oxidation (FAO) enhances histone acetylation on the Stat1 locus, thereby orchestrating the induction of T1IFN signaling in myeloid cells.

## RESULTS

### ACAT1 depletion dampens T1IFN signaling

To elucidate the role of ACAT1, we first examined its expression profile in circulating immune cells using publicly available databases. Single-cell RNA sequencing (RNA-seq) data from human peripheral blood mononuclear cells (PBMCs) were interrogated in the Immunological Genome Project database (https://immgen.org/). Analysis of the Leiden algorithm revealed that Acat1 expression is highly enriched across all PMBC lineages (fig. S1A). Moreover, data from the Human Protein Atlas (https://proteinatlas.org/) show that ACAT1 is localized to mitochondria and expressed in various PBMC cell populations, albeit at lower levels in polymorphonuclear leukocytes (fig. S1B).

**Fig. 1.**
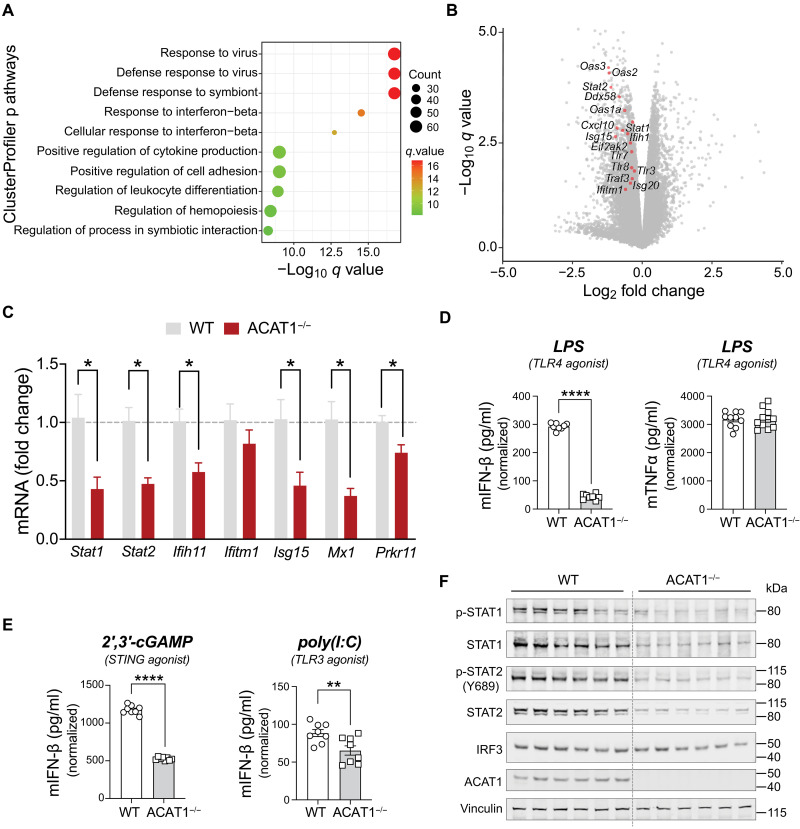
T1IFN pathway is down-regulated in ACAT1^−/−^ macrophage cells. (**A**) Dot plot of the ClusterProfiler pathway from 1618 down-genes of a total of 3288 common DE genes from the naïve cells. The *x* axis represents negative log_10_-transformed *q* values, and the dot-plot color was scaled to transformed *q* values. The size of the dot was scaled to the number of genes overlapping with the indicated pathway on the *y* axis. (**B**) Volcano plot of DE genes (*P* < 0.05) from WT versus ACAT1^−/−^ macrophage cells under basal state (*n* = 3 different lines per genotype). The statistically significant T1IFN genes are highlighted in red circles and labeled. (**C**) Quantitative RT-PCR analysis of T1IFN pathway–related genes in WT versus ACAT1^−/−^ cells (*n* = 3 lines per genotype). Data were normalized to 18*S* rRNA and represented as means ± SEM. *P < 0.05. Unpaired two-tailed Student’s *t* test. (**D**) IFN-β or TNFα production measured by ELISA from WT versus ACAT1^−/−^ cells stimulated with TLR4 agonist (*n* = 8 replicates per treatment). (**E**) IFN-β production measured by ELISA from WT versus ACAT1^−/−^ cells stimulated with STING or TLR3 agonist (*n* = 8 replicates per treatment). Data were represented as means ± SEM. **P < 0.01; ****P < 0.0001. Unpaired two-tailed Student’s *t* test. (**F**) Representative immunoblot of phospho-STAT1/2 and total STAT1/2 levels in WT versus ACAT1^−/−^ cells stimulated with LPS (*n* = 6 per genotype).

To further investigate ACAT1’s role in innate immune cells, we used the murine J774A.1 macrophage cell line and generated an isogenic cell line lacking ACAT1 via CRISPR-Cas9 gene editing. We conducted RNA-seq on both unstimulated or lipopolysaccharide (LPS)–stimulated wild-type (WT) and ACAT1 knockout (KO/ACAT^−/−^) macrophages. Principal components analysis (PCA) of differentially expressed (DE) genes showed that the presence or absence of the Acat1 gene was the principal component driving gene expression changes, irrespective of the cell activation status (fig. S2). A total of 3288 genes were DE, with 1618 genes up-regulated and 1670 genes down-regulated in unstimulated ACAT1^−/−^ cells (fig. S3A and table S1). Pathway enrichment analysis using ClusterProfiler that is curated for pathways linked to immune cell type and signaling revealed that the most significantly regulated DE genes were associated with responses to viral infections and interferon-β (IFN-β) pathway (Fig. 1A and fig. S3B).

As depicted on the volcano plot of DE genes, transcripts encoding signaling and regulatory proteins in the T1IFN pathway were robustly down-regulated following Acat1 deletion (Fig. 1B). Quantitative reverse transcription polymerase chain reaction (RT-PCR) confirmed the reduction in transcript levels of T1IFN pathway genes in ACAT1^−/−^ macrophages (Fig. 1C). To functionally validate these transcript signatures, we stimulated WT and ACAT1^−/−^ cells with various T1IFN inducers, including LPS [Toll-like receptor 4 (TLR4) agonist], 2′,3′-cGAMP [cyclic guanosine and adenine monophosphate (STING agonist)], and poly(I:C) [Polyinosinic:polycytidylic acid (TLR3 agonist)]. Here, ACAT1^−/−^ cells exhibited significantly diminished IFN-β secretion compared to WT controls (Fig. 1, D and E). The canonical TLR4-induced cytokine tumor necrosis factor–α (TNFα) was not affected in ACAT1^−/−^ cells (Fig. 1D, right panel).

Further assessment of canonical T1IFN signaling in response to LPS showed that pathway activation, including phosphorylation of STAT1 and STAT2, was markedly reduced in ACAT1^−/−^ cells. Notably, the steady-state levels of STAT1 and STAT2 were substantially lower, whereas interferon regulatory factor 3 (IRF3) levels remained unchanged (Fig. 1F). Together, these data suggest that ACAT1 plays a critical role in regulating T1IFN response, primarily through the modulation of selective gene expression.

### ACAT1 depletion attenuates histone acetylation

**Fig. 2.**
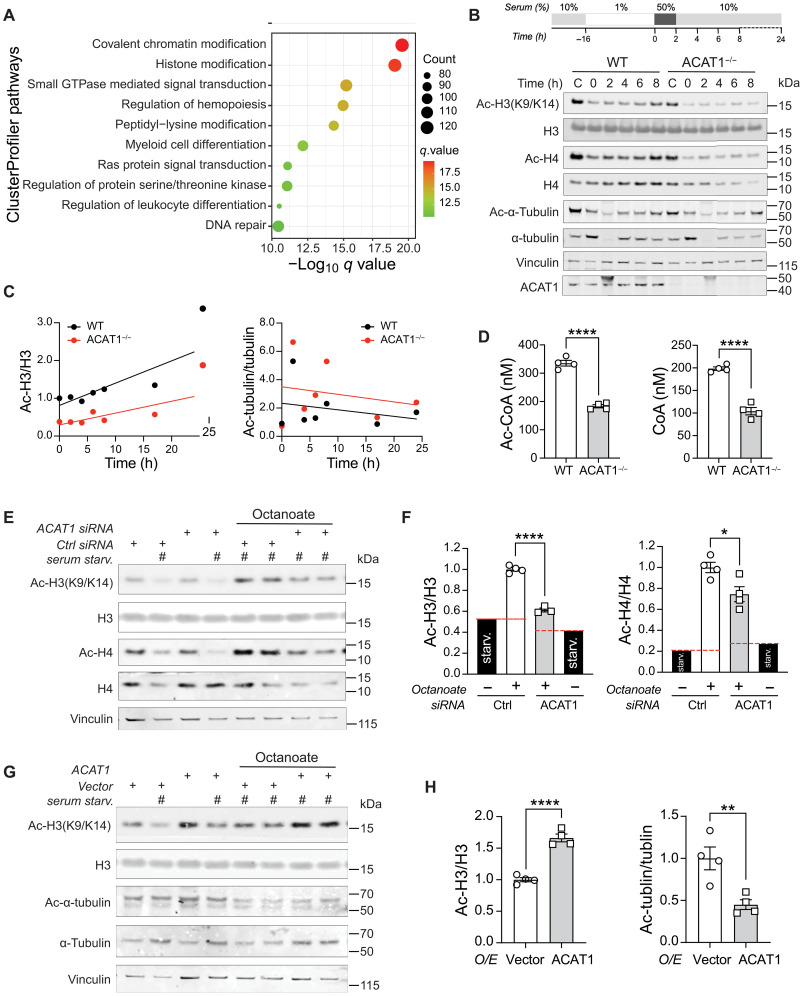
Fatty acid–mediated histone acetylation is impaired in ACAT1-deficient cells. (**A**) Dot plot of the ClusterProfiler pathway from 2760 down-regulated genes of a total of 4255 common DE genes (LPS-stimulated). The *x* axis represents negative log_10_-transformed *q* values, and the dot-plot color was scaled to transformed *q* values. The size of the dot was scaled to the number of genes overlapping with the indicated pathway on the *y* axis. (**B**) Scheme and Western blotting of histone acetylation in WT and ACAT1^−/−^ cells during cellular response to serum stimulation. h, hours. (**C**) Ratio of acetylated histone H3 to total H3 or acetylated tubulin to total tubulin was quantitated at each time point and fitted to a linear regression curve. (**D**) Acetyl-CoA or CoA level was assessed in WT and ACAT1^−/−^ cells by HPLC. (**E**) Western blotting of histone acetylation in human monocytes transfected with either control siRNA or ACAT1 siRNA under serum starvation in combination with octanoate (2 mM). (**F**) Quantitative analysis of acetylated H3 to total H3 or acetylated H4 to total H4 ratio in above siRNA-transfected cells. (**G**) Western blotting of histone acetylation in human monocytes transfected with either an empty vector or human ACAT1-expressing plasmid under serum starvation in combination with octanoate (2 mM). (**H**) Quantitative analysis of acetylated H3 to total H3 or acetylated α-tubulin to total α-tubulin ratio in above transfected cells. Data were analyzed by unpaired two-tailed Student’s *t* test. All data were represented as means ± SEM. *P < 0.05; **P<0.01; ****P < 0.0001.

ClusterProfiler enrichment analysis curated for intracellular signaling pathways also uncovered that chromatin and histone modification pathways were differentially regulated in ACAT1^−/−^ cells compared to WT counterparts upon LPS stimulation (Fig. 2A, fig. S3C, and table S2). To assess whether histone acetylation was altered in the absence of ACAT1, we used a cellular mitogenic stimulation protocol to induce a net increase in histone acetylation by synchronizing cells through serum starvation and reintroduction (shown in the inset of Fig. 2B) (*24*). Immunoblot analysis over an 8-hour period following serum stimulation indicated increased acetylation of nuclear H3 and H4 in control cells but not in ACAT1^−/−^ cells (Fig. 2B). Tubulin acetylation in the cytosol showed similar dynamics between WT and ACAT1^−/−^ cells (Fig. 2B). The temporal changes in H3 acetylation compared to tubulin over a 24-hour post-serum stimulation period are shown in Fig. 2C.

ACAT1, a distal enzyme in FAO, produces acetyl-CoA, which positively regulates histone acetylation to facilitate gene transcription (*24*, *30*). We therefore measured levels of acetyl-CoA and CoA in WT and ACAT1^−/−^ cells using high-performance liquid chromatography (HPLC). As expected, the levels of acetyl-CoA and CoA were significantly lower in the ACAT1^−/−^ cells (Fig. 2D). Beyond the murine macrophage cell line, we also used primary human monocytes with small interfering RNA (siRNA) knockdown to examine how ACAT1 regulates histone acetylation through various metabolic pathways using different upstream substrates, e.g., glucose, and the medium-chain fatty acid octanoate (Fig. 2E and fig. S4). Octanoate supplementation had the most robust effect on H3 and H4 acetylation, and these modifications were blunted following the knockdown of *ACAT1* in human monocytes (Fig. 2, E and F). In contrast, glucose-mediated histone acetylation in human monocytes was unaffected by *ACAT1* knockdown (fig. S4).

To further demonstrate the pivotal role of the FAO pathway in histone acetylation, we used siRNA to knock down two key enzymes involved in medium-chain FAO: Acyl-CoA synthetase medium chain family member 2A (ACSM2A) and Acyl-CoA dehydrogenase (ACADM) (fig. S5A). *ACSM2A* knockdown significantly blunted H3 and H4 acetylation following octanoate supplementation (fig. S5, B and C), whereas *ACADM* knockdown had no impact, likely due to its low expression in human monocytes (https://proteinatlas.org/). Conversely, overexpression of ACAT1 in the presence of octanoate increased histone H3 acetylation but did not affect the acetylation of cytoskeletal protein α-tubulin (Fig. 2, G and H).

**Fig. 3.**
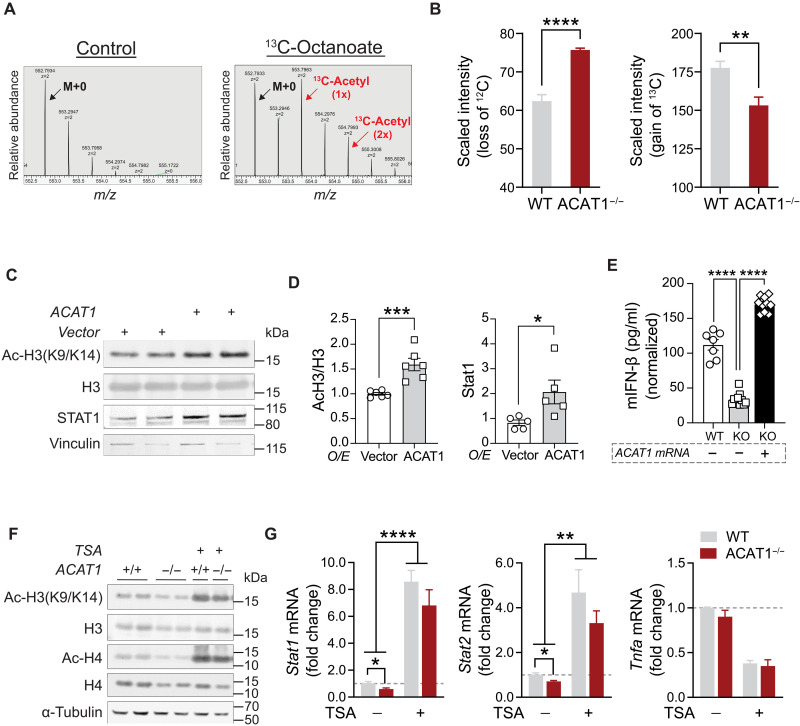
Histone acetylation is required for ACAT1-mediated transcriptional regulation of STAT1 and T1IFN production. (**A**) Representative MS spectra from WT cells treated with vehicle (left) or [U-^13^C]octanoate (right), highlighting the isotope distribution of histone H3 peptides acetylated on one or two lysine residues. Mass shifts to the right indicate the presence of [U-^13^C]octanoate-derived carbon at those specific histone lysine residues. (**B**) Bar graph showing the loss of ^12^C (left) and the gain of ^13^C (right) from the precursor ion of the singly acetylated peptide of H3K9 or H3K14. Data were represented as means ± SD. **P < 0.01; ****P < 0.0001. (**C**) Western blotting of histone H3 acetylation and STAT1 in human monocytes transfected with either an empty vector or human ACAT1-expressing plasmid under normal serum conditions. (**D**) Quantitative analysis of acetylated H3 to total H3 ratio or STAT1 level (normalized to vinculin) in above transfected cells. Data were represented as means ± SEM. *P < 0.05; ***P < 0.001. (**E**) Mouse IFN-β production measured by ELISA from WT, ACAT1^−/−^ cells, or KO cells transfected with ACAT1 mRNAs. Data were represented as means ± SEM. ****P < 0.001. (**F**) Western blotting of histone acetylation in WT and ACAT1^−/−^ cells treated with vehicle or TSA. (**G**) Quantitative RT-PCR analysis of mouse Stat1, Stat2, and Tnfα genes in WT and ACAT1^−/−^ cells treated with either vehicle or TSA for 16 hours. Data were normalized to 18*S* rRNA and represented as means ± SEM. *P < 0.05; **P < 0.01; ****P < 0.0001.

Furthermore, to determine whether histone acetylation via FA-derived acetyl-CoA is substantially impaired in ACAT1^−/−^ cells, we performed stable isotope tracing with uniformly labeled ^13^C-octanoate to track incorporation into whole-cell acetyl-CoA pools, followed by liquid chromatography–mass spectrometry detection of histone H3 K9 and K14 acetylation (Fig. 3A). The degree of ^13^C incorporation in acetyl-H3 was markedly reduced in ACAT1^−/−^ macrophage cells (Fig. 3B). Together, these findings underscore the critical role of mitochondrial ACAT1 in linking lipid-derived acetyl-CoA to nuclear histone acetylation.

### Histone acetylation is required for ACAT1-mediated up-regulation of STAT1 and T1IFN

Histone acetylation, a pivotal posttranslational modification known for facilitating gene transcription by modulating nucleosomal conformation through the dissociation of histones from DNA, plays a critical role in cellular processes. We therefore examined the impact of ACAT1 overexpression on histone acetylation and STAT1 levels in human monocytes. Here, ACAT1 overexpression led to a significant increase in H3(K9/K14) acetylation and concurrently elevated the steady-state levels of STAT1 (Fig. 3, C and D). Furthermore, reintroduction of Acat1 into ACAT1^−/−^ J774A.1 cells resulted in a concomitant induction of LPS-mediated IFN-β secretion, reaching levels comparable to those observed in WT cells (Fig. 3E). To validate the involvement of histone acetylation in T1IFN biology, we then used trichostatin A (TSA), a broad histone deacetylase (HDAC) inhibitor. Here, TSA treatment markedly augmented histone H3 and H4 acetylation in both WT and KO macrophages without altering total protein levels (Fig. 3F). In addition, TSA increased transcript levels of Stat1 and Stat2 but did not affect Tnfα expression in either WT or ACAT1^−/−^ cells (Fig. 3G).

**Fig. 4.**
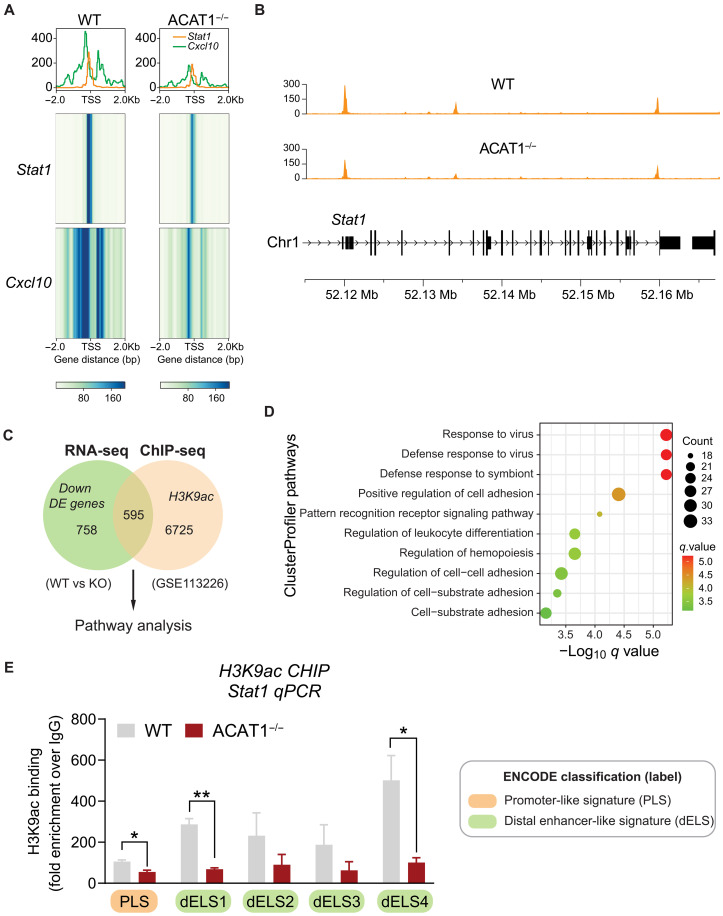
Chromatin accessibility and binding of acetylated histone H3 to the *Stat1* locus are reduced in ACAT1^−/−^ cells. (**A**) Heatmap and normalized signal density in aligned TSS and flanking regions of *Stat1* and *Cxcl10* genomic loci in WT versus ACAT1^−/−^ cells. The one-dimensional signal at the top of the figure is the column summation of the bottom heatmap plot. The value in the top plots is the RPGC. (**B**) ATAC-seq tracks at the *Stat1* locus in LPS-stimulated WT and ACAT1^−/−^ cells, showing the normalized coverage and genomic context of the entire *Stat1* gene cassette. (**C**) Flowchart showing down-regulated DE genes from the naïve RNA-seq dataset and H3K9ac ChIP-seq dataset (GSE113226, unstimulated cells) used to identify DE genes specifically regulated by ACAT1 and H3K9 acetylation. The 595 overlapping DE genes were subjected to pathway enrichment analysis. (**D**) Dot plot of the ClusterProfiler pathway from above 595 overlapping DE genes of a total of 2969 common DE genes. The *x* axis represents negative log_10_-transformed *q* values, and the dot-plot color was scaled to transformed *q* values. The size of the dot was scaled to the number of genes overlapping with the indicated pathway on the *y* axis. (**E**) Quantitative H3K9ac ChIP-PCR analysis of mouse Stat1 promoter/enhancer sequences in WT and ACAT1^−/−^ cells. IgG, immunoglobulin G. Data were represented as means ± SEM. *P < 0.05; **P < 0.01. Unpaired two-tailed Student’s *t* test.

To further investigate the mechanism by which ACAT1 regulates the transcription of T1IFN-related genes, we performed assay for transposase-accessible chromatin using sequencing (ATAC-seq) in WT and ACAT1^−/−^ cells following LPS stimulation. The ATAC-seq pathway enrichment analysis indicates that chromatin accessibility is differentially regulated between WT and ACAT1^−/−^ cells across various immune response signaling pathways (fig. S6). Notably, peak coverage for genes in the IFN-α/β signaling pathway is reduced in the KO cells compared to WT cells (fig. S7). Normalized coverage around the transcription start site (TSS) for *Stat1* and interferon-stimulated gene *Cxcl10* is substantially higher in WT cells than in KO cells, indicating diminished chromatin accessibility at the *Stat1* and *Cxcl10* loci in the KO cells (Fig. 4, A and B). Before delving deeper, we used an integrated bioinformatics approach to compare down-regulated DE genes in our RNA-seq dataset (WT versus ACAT1^−/−^) with a publicly available H3K9ac chromatin immunoprecipitation sequencing (ChIP-seq) dataset from naïve WT murine bone marrow–derived macrophages (GSE113226). The Venn diagram illustrates an overlap of 595 genes between these datasets (Fig. 4C). Subsequent ClusterProfiler pathway analysis supported a notable overlap of genes associated with the antiviral response in these datasets (Fig. 4D). To examine potential modifications in the transcriptional machinery of Stat1 in the presence or absence of ACAT1, we used the UCSC genome browser (*28*) to annotate the promoter and enhancer loci of Stat1 (fig. S8). Quantitative H3K9ac ChIP-murine Stat1-PCR revealed that the deletion of Acat1 resulted in diminished H3K9ac binding at the putative promoter and selective distal enhancers of the murine Stat1 gene (Fig. 4E). These findings suggest that ACAT1 could influence Stat1 gene expression through histone acetylation and its binding to the upstream regulatory elements of the Stat1 locus.

### The carnitine shuttle is essential for ACAT1-mediated histone H3 acetylation

**Fig. 5.**
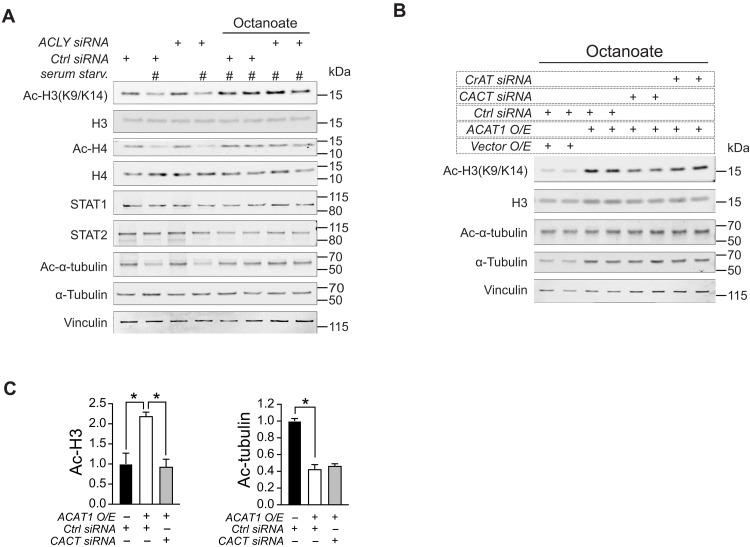
Carnitine shuttle is required for the action of ACAT1 on histone acetylation. (**A**) Western blotting of histone acetylation, STAT1, and STAT2 in human monocytes transfected with either control siRNA or ACLY siRNA under serum starvation in combination with octanoate (2 mM). (**B**) Western blotting of histone and tubulin acetylation in human monocytes transfected with either an empty vector and control siRNA or ACAT1 and/or control siRNA, CACT siRNA, and CrAT siRNA under serum starvation in combination with octanoate (2 mM). (**C**) Quantitative analysis of acetylated H3 to total H3 or acetylated α-tubulin to total α-tubulin ratio in above transfected cells. Data were represented as means ± SEM. *P < 0.05. One-way ANOVA followed by Dunnett’s multiple comparisons test.

Acetyl-CoA, a crucial substrate for histone acetylation, cannot directly traverse organelle membranes, necessitating a transport mechanism across mitochondrial membranes for FAO-mediated nuclear-cytosolic acetylation events. The pathways governing histone acetylation from mitochondrial-derived acetyl-CoA have been shown to be dependent (*24*) or independent on adenosine triphosphate–citrate lyase (ACLY) (*25*, *31*). In the latter case, recent studies suggest that both glucose and fatty acids can supply nuclear-cytosolic acetyl-CoA through a pathway that does not require ACLY but involves acetylcarnitine shuttling (*24*, *25*). We sought to understand which cellular mechanism is involved in the ACAT1-mediated histone acetylation from FA-derived carbon. Unlike ACAT1, the siRNA knockdown of ACLY did not disrupt histone H3 and H4 acetylation in human monocytes following overnight serum starvation and subsequent treatment with octanoate (Fig. 5A). We then targeted various components of acetylcarnitine shuttling, including carnitine acetyltransferase (CrAT) and the CACT. Following overexpression of ACAT1 in human monocytes, siRNA knockdown of *CrAT* resulted in a modest reduction, whereas knockdown of *CACT* led to a more pronounced decrease in histone H3 acetylation without altering α-tubulin acetylation levels (Fig. 5, B and C). These findings suggest that acetyl-CoA generated through mitochondrial ACAT1 is reliant on the acetylcarnitine shuttle for histone acetylation.

### ACAT1 and histone acetylation are induced in monocytes from obese compared to lean human subjects

**Fig. 6.**
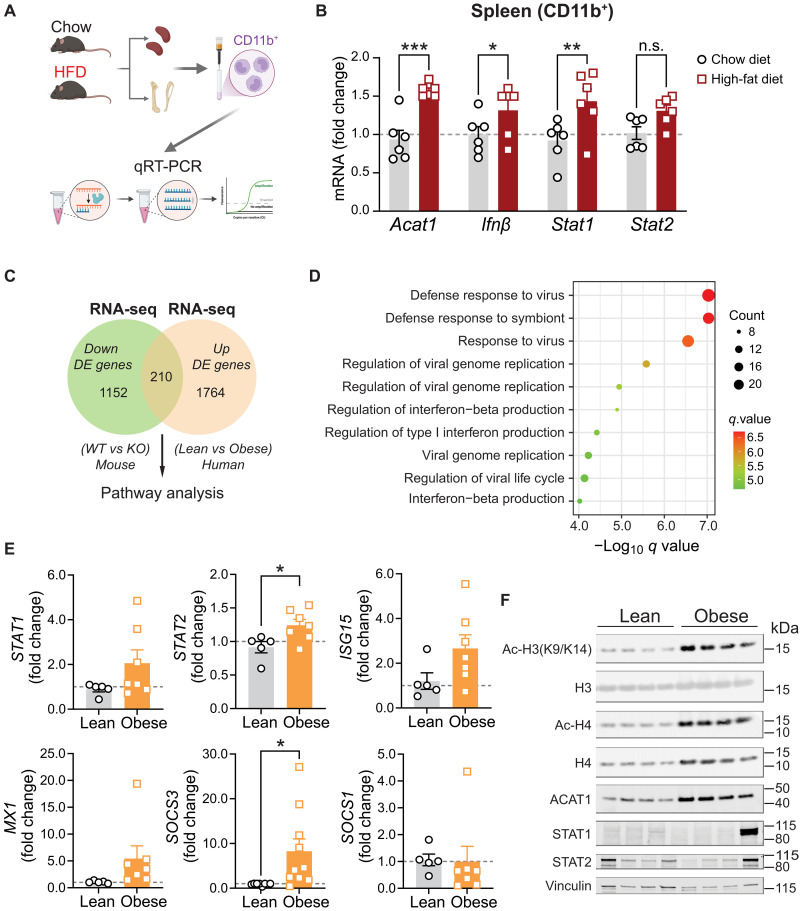
ACAT1 and T1IFN genes levels are elevated in HFD-fed mice and in human subjects with obesity. (**A**) Scheme of the HFD mouse study on gene expression. (**B**) Quantitative RT-PCR analysis of *Acat1* and T1IFN pathway–related genes in CD11b^+^ cells isolated from the mouse spleen fed with chow diet or HFD (*n* = 6 mice per group). Data were normalized to 18*S* rRNA and represented as means ± SEM. Two-way ANOVA followed by Tukey’s multiple comparisons test. *P < 0.05; **P < 0.01; ***P < 0.001. n.s., not significant. (**C**) Flowchart showing an overlap of 210 genes from down-regulated DE genes in the ACAT1^−/−^ RNA-seq dataset (unstimulated) and up-regulated DE genes from the obese PBMC RNA-seq dataset (unstimulated) used to identify pathways specifically regulated by ACAT1 and obesity. (**D**) Dot plot of pathway analysis from above 210 overlapping DE genes of a total of 2969 common DE genes. The *x* axis represents negative log_10_-transformed *q* values, and the dot-plot color was scaled to transformed *q* values. The size of the dot was scaled to the number of genes overlapping with the indicated pathway on the *y* axis. (**E**) Quantitative RT-PCR analysis of T1IFN pathway–related genes in monocytes from lean and obese subjects (*n* = 4 to 5 subjects per group). Data were normalized to 18*S* rRNA and represented as means ± SEM. *P < 0.05. Unpaired two-tailed Student’s *t* test. (**F**) Immunoblot of histone acetylation and ACAT1 in lean versus obese monocytes (*n* = 4 subjects per lean or obese group).

Given the association of obesity with disrupted lipid metabolism and low-level inflammation, we aimed to investigate whether the T1IFN pathway, modulated by ACAT1, is constitutively activated by obesity. To investigate this, we first assessed the transcript levels of *Acat1* and interferon-stimulated genes (*ISGs*) in splenic and bone marrow–derived CD11b^+^ myeloid cells, comparing a 14-week standard chow diet with a 60% high-fat diet (HFD). The transcript level of Stat1 was substantially elevated in both populations of myeloid cells (Fig. 6, A and B, and fig. S9A). In addition, *Acat1* and several *ISGs* were up-regulated in splenic CD11b^+^ myeloid cells (Fig. 6B). To connect our mouse study with human obesity, we used integrative bioinformatics by comparing DE genes between WT and ACAT1^−/−^ murine macrophage, translated into a human database (*32*), with an RNA-seq dataset comparing PBMC gene expression in lean versus obese subjects (*33*). The analysis revealed 210 overlapping DE genes, with blunted expression in ACAT1^−/−^ cells and induced expression in obese PBMCs (Fig. 6C). Pathway analysis of these overlapping DE genes highlighted significant divergence in genes associated with T1IFN signaling (Fig. 6D).

In a cohort of lean and obese (fig. S9B), otherwise healthy, subjects recruited under our Human Disease Discovery Protocol (NCT01143454), research blood was collected to isolate primary monocytes using negative selection. Quantitative RT-PCR analysis of these monocytes revealed modest induction of transcripts related to the T1IFN pathway in obese subjects, including the expression of *STAT1*, *CXCL10*, *ISG15*, *MX1*, and *SOCS3* (Fig. 6E). Immunoblot analysis on monocytes from the same subjects showed that obesity was associated with increased ACAT1 levels and elevated histone H3 and H4 acetylation (Fig. 6F). Collectively, these findings suggest that ACAT1-mediated histone acetylation on the transcriptional regulation of T1IFN pathway could be operational in patients with obesity.

## DISCUSSION

In this study, we uncover that acetyl-CoA, generated through mitochondrial FAO, plays a pivotal regulatory role in the induction of T1IFN signaling in both murine and human macrophages. This effect is dependent on mitochondrial ACAT1, the CACT, and histone acetylation. Histone acetylation, in turn, up-regulates the canonical T1IFN signaling pathway and the transcriptional regulatory mediator STAT1. Furthermore, preliminary data suggest that this program may be induced and functional in mice in response to a HFD and as a component of low-grade inflammation in human subjects with obesity (*34*).

The concept that metabolic pathways play a critical role in innate and adaptive immunity is well established and described as immunometabolic regulation. Evidence supports the immunomodulatory roles of individual metabolites functioning as signaling intermediates, including those from the tricarboxylic acid (TCA) cycle, purine pathway, amino acid metabolites, short-chain fatty acids, and ketones (*3*, *10*, *35*–*37*). In this context, acetyl-CoA is emerging as an important immunomodulatory metabolite in its role as a mediator of histone acetylation downstream of glucose and fat catabolism (*24*, *25*). Glucose-induced histone acetylation is orchestrated by its oxidative metabolism to generate citrate in the TCA cycle. Citrate is then transported to the cytosol and the nucleus, where it is converted by ACLY into acetyl-CoA, facilitating subsequent histone acetylation.

In the context of immunometabolism, TLR4 activation via MyD88 and TRIF increases glucose oxidation–dependent, ACLY-mediated histone acetylation to promote LPS-inducible gene sets in murine macrophages (*38*). Furthermore, in a human macrophage transformed cell line, cytokine production driven by the cyclic GMP-AMP-synthase (cGAS)-stimulator of interferon genes (STING) and TANK-binding kinase 1 (TBK1) pathways is dependent on glucose oxidation and ACLY levels (*39*). ^13^C-tracing studies in hepatocytes show that the oxidation of the medium-chain fatty acid octanoate becomes the preferential substrate for histone acetylation, at the expense of glucose and glutamine catabolism (*31*). That study also demonstrated that octanoate-driven histone acetylation is independent of ACLY (*31*). Recently, acetylcarnitine shuttling has been implicated as a mechanism for transferring two-carbon units from the mitochondria to the cytosol to support histone acetylation (*25*). In our study, we further define this pathway by showing that, in macrophages, the CACT and, to a lesser extent, CrAT are required for this octanoate-directed histone acetylation. Moreover, we demonstrate that this system is operational in the induction of T1IFN in macrophages and that this process is dependent on ACAT1 in the FAO pathway. Together, emerging data indicate that distinct metabolic programs orchestrate specific histone acetylation signatures. This observation raises an intriguing question regarding what are the underlying mechanisms. We believe that this remains uncharacterized at present. However, further integration of ATAC-seq and RNA-seq analysis, in the context of preferential utilization of metabolic pathways and the activation of immune cells driven by different receptors, should enhance our understanding of this biology.

The role of lipids in immunometabolism has predominantly been evaluated in the context of arachidonic acid and prostaglandins effects (*19*, *40*, *41*), as well as the immunomodulatory roles of specific lipid species, including saturated (*42*) and short-chain fatty acids (*36*). In addition, FAO itself has been shown to be necessary for the integrity and function of CD8^+^ memory T cells (*43*) and CD4^+^ regulatory T cells (*44*). This study expands on this body of work by demonstrating the importance of FAO in macrophages for controlling inflammatory gene histone acetylation, specifically through the regulation of STAT1, which subsequently transactivates T1IFN signaling. Further work will be needed to evaluate putative distinct roles of different fatty acid species in this regulation.

ACAT1 has pleiotropic substrates, playing a role in the catabolism of fatty acids, isoleucine, and ketone bodies. Furthermore, mitochondrial ACAT1 facilitates the acetylation of components of the pyruvate dehydrogenase complex to modulate glucose homeostasis (*26*). In that study, ACAT1-mediated acetylation of these components inhibited the complex activity, promoting glycolysis over glucose oxidation and thus functionally supporting tumor cell proliferation (*26*). Although not yet established, this mechanism of impeding glucose oxidation could potentially also be operational to promote the preferential use of fatty acids for histone acetylation in the presence of octanoate.

In conclusion, this study contributes to the emerging body of evidence that various aspects of lipid metabolism and different lipid metabolites play important roles in the regulation of immune function. Furthermore, our findings reveal that FAO may have an important epigenetic role in myeloid cells in controlling T1IFN signaling. In addition, our preliminary data comparing monocytes in lean and obese individuals suggest that this program may possibly be operational in nutrient-level linked immunomodulation in human disease pathophysiology.

## MATERIALS AND METHODS

### Generation of ACAT1^−/−^ murine macrophage J774A.1 cell line

WT and ACAT1^−/−^ cells were generated by transfecting a pSpCas9-2A-GFP vector with no guide RNA or containing a guide RNA targeting the fourth exon of murine Acat1 gene. Following transfection, cells were sorted by fluorescence-activated cell sorting (FACS) based on green fluorescent protein (GFP) fluorescence and plated to a 96-well plate at a density of ~5 cells per well (not all sorted cells may survive the FACS process or the subsequent outgrowth; therefore, the number of cells sorted was adjusted to five instead of one). Single-cell clonal populations for both WT and ACAT1^−/−^ were generated and expanded for 2 to 3 weeks. The indel mutation at the target loci was verified by genomic DNA sequencing. The protocol was followed as previously published (*45*).

### RNA-seq and bioinformatics analysis

Total RNA was extracted with the NucleoSpin RNA kit (Takara), and RNA integrity was assessed by the Agilent Technologies 2100 Bioanalyzer (Agilent). Dual-index libraries were constructed with at least one unique index per each library using the NEB Next Ultra II Directional RNA Library Prep Kit for Illumina to enable subsequent pooling of equal quantities of individual libraries. The integrity and ratio of pooled libraries was validated using the MiSeq system (Illumina), and then paired-end sequencing [2×100 base pairs (bp)] was performed on an NovaSeq 6000 sequencer (Illumina) with the Illumina an NovaSeq 6000 Kit by the DNA Sequencing and Genomics Core at the National Heart, Lung, and Blood Institute (NHLBI).

FastQC (www.bioinformatics.babraham.ac.uk/projects/fastqc/) was used to confirm quality of RNA-seq fasta files. Trimmomatic tool (V0.33; https://github.com/timflutre/trimmomatic) was used to remove adapters, followed by quality trimming and alignment to the mouse genome (GRCm38) using HISAT2.40 (https://daehwankimlab.github.io/hisat2/) (*46*). Gene expression levels were quantified using StringTie (https://github.com/gpertea/stringtie) (*46*, *47*). DE genes were evaluated using Ballgown (http://bioconductor.org/packages/release/bioc/html/ballgown.html) (*48*). Genes with a *P* value of <0.05 were considered DE genes. Pathway enrichment analysis was performed using the R package clusterProfiler56 on DE genes that were subset into up-regulated and down-regulated based on the fold change values.

### ATAC-seq and bioinformatics analysis

Thirty thousand LPS-stimulated WT and ACAT1^−/−^ J774A.1 cells were used in the experiment. Each cell was subjected to tagmentation reaction and purification using the ATAC-Seq Kit (Active Motif no. 53150) according to the manufacturer’s instructions. Tagged DNA was amplified by 10 cycles of PCR with an index primer unique to each sample. The libraries were quantified by the TapeStation System (Agilent) and sequenced on the Illumina NovaSeq X instrument using 50-bp paired-end reads to a depth of >50 million mapped reads.

The OpenOmics chrom-seek pipeline (version 1.2.0; https://github.com/OpenOmics/chrom-seek) was used to process the ATAC-seq data starting from raw FastQ files to assess quality and evaluate chromatin accessibility. Internally, this pipeline used cutadapt (version 1.18) (*49*) to trim adapter sequences and bwa-mem (version 0.7.17) (*50*) and to align the trimmed reads to the GRCm38 mouse reference genome (*51*). Any reads aligning to ENCODE blacklist regions were removed. PCR duplicates were marked using Picard (MarkDuplicates) (Broad, 2024). PCR duplicates and alignments with a mapping quality (mapQ) score of ≤5 were removed. Chromatin accessibility peaks were called using Genrich (version 0.6; https://github.com/jsh58/Genrich), and differential peak analysis was performed using DiffBind (version 3.19) (*52*). Peaks were annotated with uropa to identify all protein coding genes within 100 kb of the peak midpoint and then were collapsed down to the closest gene feature for downstream analysis. Annotated peaks with an adjusted *P* value of ≤0.05 were considered significant. Enriched pathways were identified via an overrepresentation analysis using the R package clusterProfiler (version 4.13.3) (*53*). Reads per genomic content (RPGC) normalization was performed to create bigwig files, and genomic regions of interest (e.g., interferon signaling) were inspected and visualized for chromatin accessibility using deeptools (version 3.5.5) (*54*).

### Human monocyte cultures

The NHLBI Institutional Review Board approved this study (NCT01143454). Lean and obese, otherwise healthy, volunteers signed informed consent before donating blood for analysis. Primary PBMCs were isolated from human blood by density centrifugation using the Lymphocyte Separation Medium (MP Biomedicals). Human monocytes were negatively selected from PBMCs using the Monocyte Isolation Kit (Miltenyi Biotec). Monocytes were then plated 0.15 × 10^6^ per well onto a 96-well plate [for enzyme-linked immunosorbent assay (ELISA)] or 10^6^ per well onto 12-well plates (for RNA isolation or Western blotting) in RPMI media supplemented with 10% human serum. Human whole blood and elutriated monocytes were obtained from the blood bank of National Institutes of Health.

### Inhibitors, siRNA, and nucleofection of human monocytes

HDAC inhibitor TSA (Cayman) was used in cell culture at 50 μM for 16 hours before subsequent assays. ON-TARGETplus siRNA for knocking down gene expression of *ACAT1*, *ACLY*, *CRAT*, *CACT*, or nontargeting control siRNA was purchased from Horizon Discovery. siRNA against human genes or control siRNA was incubated with a mixture of a nucleofection solution (Human Monocyte Nucleofector Kit, Lonza) and primary human monocytes and placed in nucleofection cuvettes subjected to program Y-010 for the Nucleofector 2b Device (Lonza). A 500-μl RPMI medium was immediately added into cuvettes after nucleofections. Cells were then plated in a 12-well plate and incubated at 37°C under 5% CO_2_ for 48 hours before harvesting for the assays. Plasmid transfections using an empty vector (pcDNA3.1) or human ACAT1-expressing constructs were conducted with the same protocol as siRNA transfection.

### RNA isolation and quantitative PCR analysis

Total RNA was extracted from monocytes using the NucleoSpin RNA kit (Takara), and RNA concentration was measured using the NanoDrop spectrophotometer (Thermo Fisher Scientific). cDNA was synthesized with the SuperScript III First-Strand Synthesis System for RT-PCR (Thermo Fisher Scientific) according to the manufacturer’s instructions. Quantitative real-time PCR was performed using the FastStart Universal SYBR Green Master (Rox) (Roche Holding) and run on LightCycler 96 Systems (Roche Holding). Transcript levels of STAT1, STAT2, and 18*S* rRNA were measured using validated gene-specific primers (QIAGEN). Primers for *ACAT1*, *ACLY*, *CRAT*, *CACT*, *ACSM2A*, and *ISGs* were custom synthesized at IDT Inc. Relative gene expression was quantified by normalizing cycle threshold values with 18*S* rRNA using the 2^−ΔΔCt^ cycle threshold method.

### Western blotting

Human monocytes were lysed using a radioimmunoprecipitation assay buffer supplemented with protease inhibitor cocktail (Roche) and phosphatase inhibitors (Sigma-Aldrich). The lysates were separated by NuPAGE 4–12% Bis-Tris Protein Gels (Thermo Fisher Scientific) and transferred to nitrocellulose membranes using the Trans-Blot Turbo Transfer System (Bio-Rad Laboratories) according to the manufacturer’s instructions. Membranes were blocked with a 50% Odyssey Blocking Buffer in PBS-T [0.1% Tween 20 in phosphate-buffered saline (PBS)] buffer and incubated with appropriate antibodies overnight at 4°C. Antibodies used included the following: STAT1, phospho-STAT1(Y701), STAT2, Acetyl-Histone H3 (Lys9/Lys14), Histone H3, Histone H4 (Cell Signaling Technology); phospho-STAT2 (Y689), Acetyl-Histone H4 (Lys5/Lys8/Lys12/Lys16) (Millipore); IRF3 (Abcam); and ACAT1 and vinculin (Sigma-Aldrich). Primary antibody incubations were followed by incubation with IRDye Secondary antibodies for 1 hour at room temperature. Immunoblots were visualized and imaged using the Odyssey CLx Imaging System (LI-COR Biosciences). Protein band intensity was measured using the ImageJ software and normalized to vinculin.

### Cell stimulation and cytokine assays

WT and ACAT1^−/−^ J774A.1 cells were incubated at 0.08 × 10^6^ cells per well in a 96-well plate in complete Dulbecco’s modified Eagle’s medium (DMEM) [10% fetal bovine serum (FBS) in DMEM] stimulated with LPS (100 ng/ml; Ultrapure Salmonella minnesota R595; Enzo Life Sciences), 2′,3′-cGAMP (2 μg/ml; InvivoGen), or poly(I:C) (2 μg/ml; InvivoGen) for 16 hours. Cell culture supernatants were collected, centrifuged to remove cells and debris, and stored at −80°C for later analysis. Cytokines of mouse IFN-β and TNFα were assayed by ELISA (R&D Systems). Results were normalized to cell number, as determined by the CyQuant cell proliferation assay (Invitrogen).

### Acetyl-CoA and CoA analysis by HPLC

WT and ACAT1^−/−^ cells were plated at a density of 5 × 10^6^ cells per 10-cm culture dish and cultured in DMEM (10% FBS) for 1 day. Cells were washed with PBS and detached from the dish using a CellStripper Solution. The cell pellet was collected by centrifugation, washed twice with PBS, and resuspended in 100 μl of a 5% 5-sulfosalicylic acid (Sigma-Aldrich) solution. To permeabilize the cells, samples were frozen in liquid nitrogen and thawed on ice. This freeze-thaw cycle was repeated twice. After centrifugation, the supernatant was filtered using the Ultrafree-MC LH Centrifugal Filter (Millipore, Billerica, Massachusetts, UFC30LH25). Then, the samples were transferred into SUN-SRi Glass Microsampling Vials (Thermo Fisher Scientific, 14-823-359) with SUN-SRi 11 mm Snap Caps (Thermo Fisher Scientific, 14-823-379), and 80 μl of each sample was separated using an Agilent 1100 HPLC (Agilent Technologies, Santa Clara, California) equipped with a reversed-phase column, Luna 3 μm C18(2) 100 Å, 50 by 4.6 mm, 3 μm (Phenomenex, Los Angeles, California). The detailed protocol was described previously (*55*).

### Stable isotope labeling

WT and ACAT1^−/−^ cells were seeded at a density of 6 × 10^6^ cells per 10-cm culture dish in DMEM containing 10% FBS. The following day, the medium was changed to DMEM containing 1% dialyzed FBS or 1% dialyzed FBS supplemented with 2 mM [U-^13^C_8_]-octanoate (Cambridge Isotope Laboratories). After 24 hours, cells were washed with ice-cold Dulbecco’s PBS (DPBS) and collected into tubes. The cells were further washed twice with ice-cold DPBS, and the cell pellet was frozen at −80°C until extraction.

### Histone purification and chemical labeling of histone peptides

Core histones were purified from cultured cells using the “Histone Purification Mini Kit” (Active Motif, Carlsbad, CA) according to the manufacturer’s instructions. The purified histones were desalted using the Zeba spin columns (Thermo Fisher Scientific, CA) prior to downstream procedures. The purity of the core histones was checked by running an SDS–polyacrylamide gel electrophoresis gel. An aliquot of 10 mg of purified core histones was labeled using the hybrid chemical derivatization method as described previously (*56*). Peptides were desalted using C18 spin columns (Thermo Fisher Scientific, CA) according to the manufacturer’s protocol. The samples were vacuum centrifuged to dryness and stored at −80°C until analysis by mass spectrometry (MS).

### MS acquisition and analysis

The dried peptide fractions were reconstituted in 0.1% trifluoroacetic acid and subjected to nanoflow liquid chromatography (Thermo Ultimate 3000 RSLCnano LC System, Thermo Fisher Scientific) coupled to an Orbitrap Eclipse mass spectrometer (Thermo Fisher Scientific, CA). Peptides were separated using a low pH gradient using 0 to 50% acetonitrile (ACN) over 60 min in mobile phase containing 0.1% formic acid at 300 nl/min flow rate. MS scans were performed in the Orbitrap analyzer at a resolution of 120,000 with an ion accumulation target set at 4 × 10^5^ and maximum injection time (IT) set at 50 ms over a mass range of 200 to 1400 mass/charge ratio (*m*/*z*). Ions with determined charge states between 2 and 6 were selected for MS2 scans. A cycle time of 3 s was used, and a quadrupole isolation window of 0.4 *m*/*z* was used for tandem mass spectrometry (MS/MS) analysis. An Orbitrap at 15,000 resolutions with a normalized automatic gain control (AGC) set at 100 followed by maximum IT set as “Auto” with a normalized collision energy setting of 30 was used for MS/MS analysis. The data analysis was performed using Skyline (*57*). The selection of the precursor and their fragment ions was automatically performed by the program. Each selected peak was manually verified, and the only the peak values for the precursor ions was retained for further analysis.

### HFD mouse study

Animal studies used in this protocol were approved by the NHLBI Animal Care and Use Committee (protocol no. H-0222R5). Six 14-week-old male C57BL/6J diet induced obesity (DIO) mice (strain no. 380050) and C57BL/6J DIO Control mice (strain no. 380056) were purchased from the Jackson Laboratory. The mice were maintained on a 12-hour/12-hour light/dark cycle and housed three mice per cage with unrestricted access to water and either 60 kcal % (Research Diets, D12492i) or 10 kcal % fat diet (Research Diets, D12450Ji) for an additional 2 weeks. The mice were then euthanized. CD11b^+^ cells were isolated from the splenocytes and bone marrow using the EasySep Mouse CD11b^+^ Positive Selection Kit (STEMCELL Technologies, catalog no. 18970A) following the manufacturer’s protocol.

### Statistical analysis

Statistical analysis was performed using the Prism 10 software (GraphPad), and results are represented as means ± SEM. unless otherwise indicated. Comparisons of two groups were calculated using paired or unpaired two-tailed Student’s *t* test. Comparisons of more than two groups were calculated using one-way analysis of variance (ANOVA) followed by multiple comparisons test (Dunnett’s or Tukey’s). Two-way ANOVA was used if there were two independent variables. For all tests, *P* < 0.05 was considered significant.
