# Supplementary material for: Mitochondrial fatty acid oxidation regulates monocytic type I interferon signaling via histone acetylation
Source: Sci Adv. 2025 Jan 22;11(4):eadq9301. doi: 10.1126/sciadv.adq9301 (PMC11753372; doi:10.1126/sciadv.adq9301)
Supplement: Supplementary file 1 — Figs. S1 to S10 Legends for tables S1 and S2 Tables S3 to S5 [file sciadv.adq9301_sm.pdf]

Science Advances
AAAS

Supplementary Materials for

Mitochondrial fatty acid oxidation regulates monocytic type I interferon signaling via histone acetylation

Jing Wu *et al.*

Corresponding author: Michael N. Sack, sackm@nih.gov

Sci. Adv. **11**, eadq9301 (2025)
DOI: 10.1126/sciadv.adq9301

The PDF file includes:

Figs. S1 to S10
Legends for tables S1 and S2
Tables S3 to S5

Other Supplementary Material for this manuscript includes the following:

Tables S1 and S2

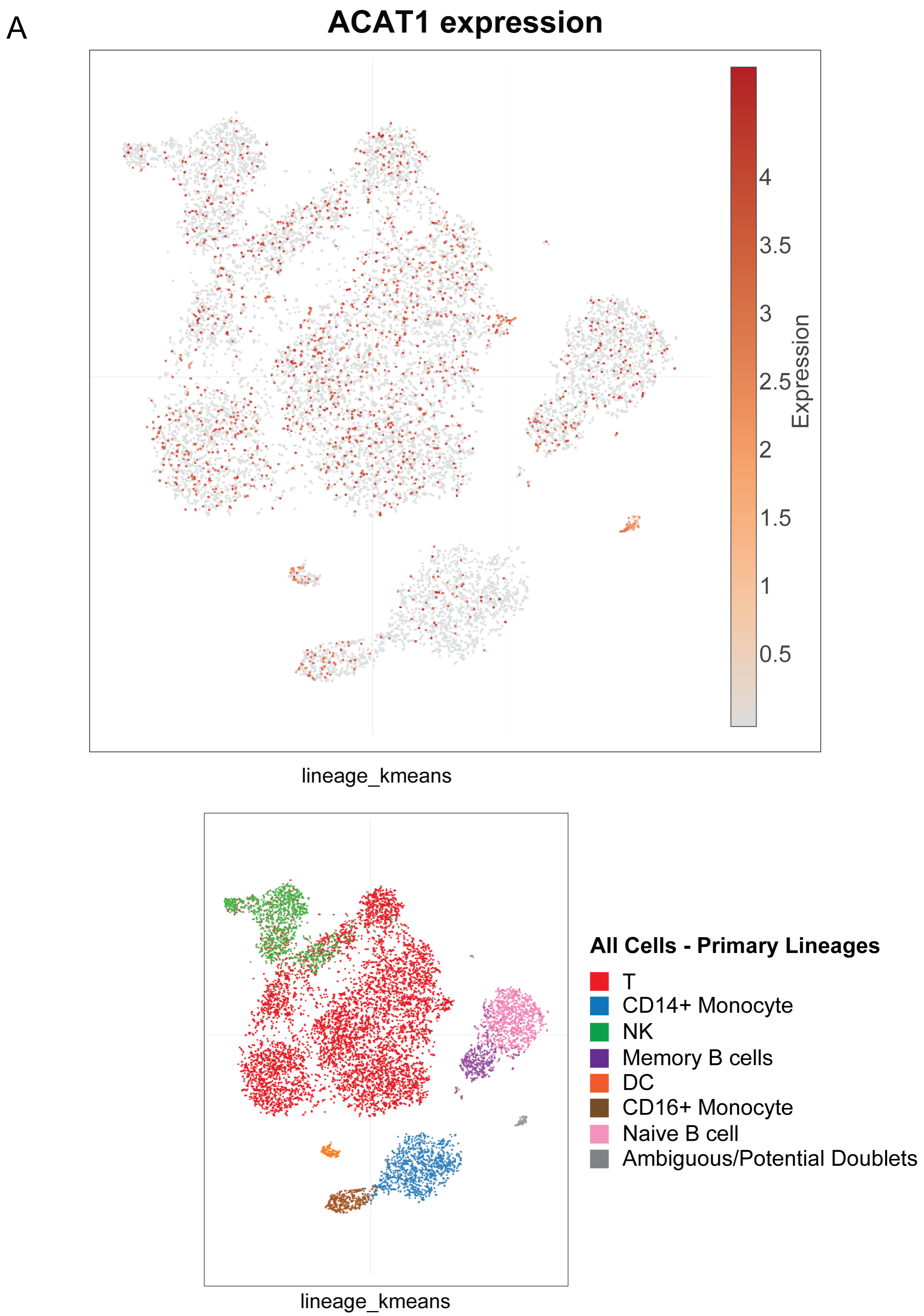
A
ACAT1 expression
4
3.5
3
2.5
2
1.5
1
0.5
Expression
lineage_kmeans
All Cells - Primary Lineages
T
CD14+ Monocyte
NK
Memory B cells
DC
CD16+ Monocyte
Naive B cell
Ambiguous/Potential Doublets
lineage_kmeans

B

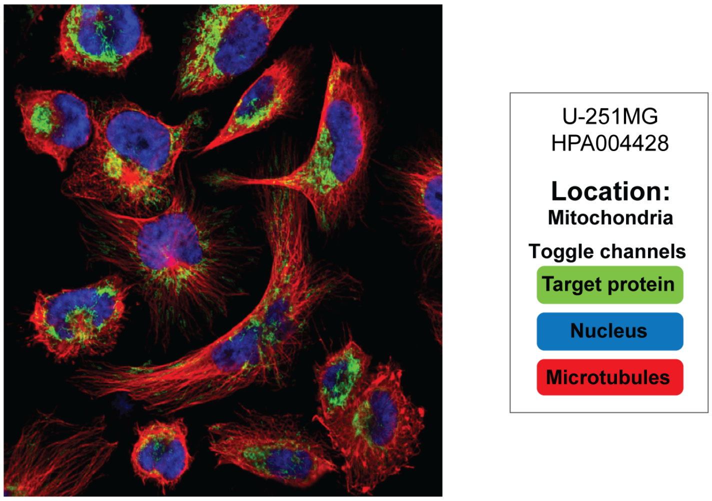

Fig. S1. Characteristics of human ACAT1 obtained from public databases.
(A) Human PBMC single cell RNA-seq datasets showing ACAT1 is ubiquitously expressed in all the primary lineages of immune cells. The resource link could be accessed at https://www.immgen.org/ (B) Human Protein Atlas project showing the cellular localization of ACAT1 in mitochondria of various cell types. An example of its localization in U-251 MG cell is shown here. The resource link and more information could be accessed at https://www.proteinatlas.org/

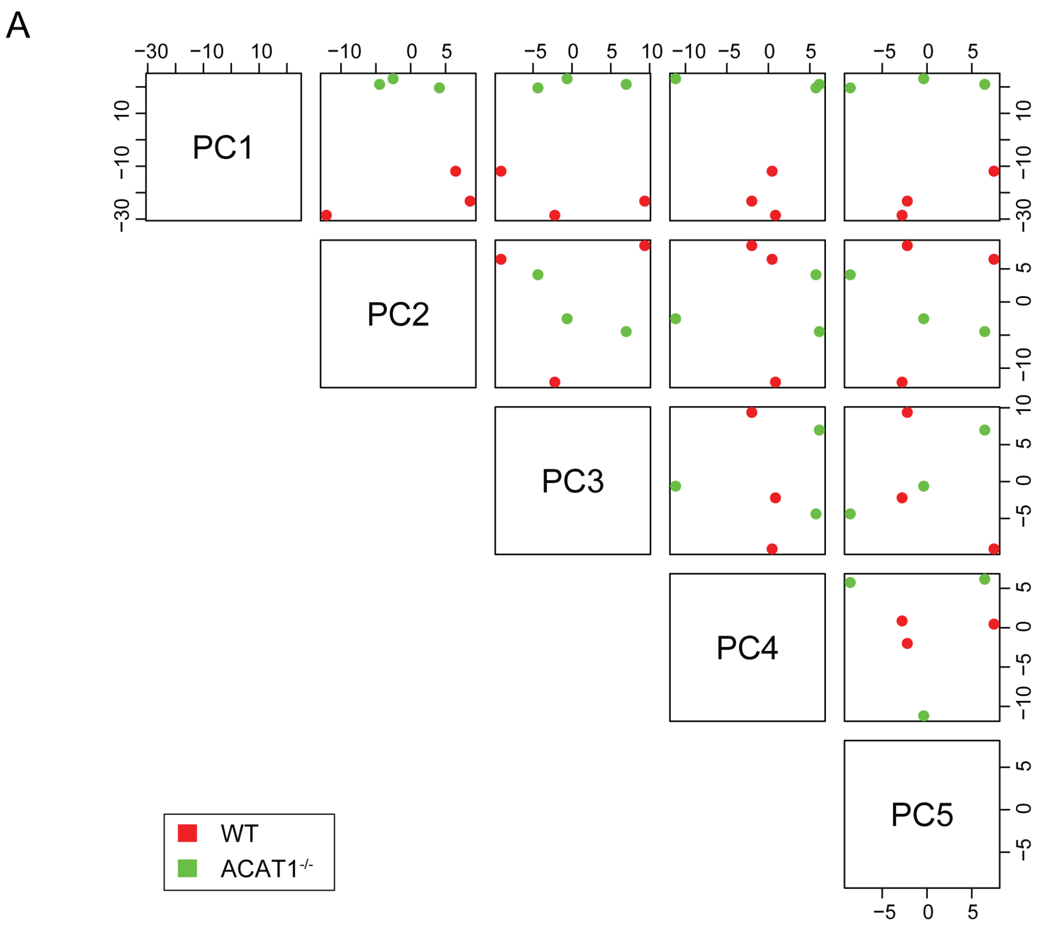

A
PC1
PC2
PC3
PC4
PC5
WT
ACAT1-/-

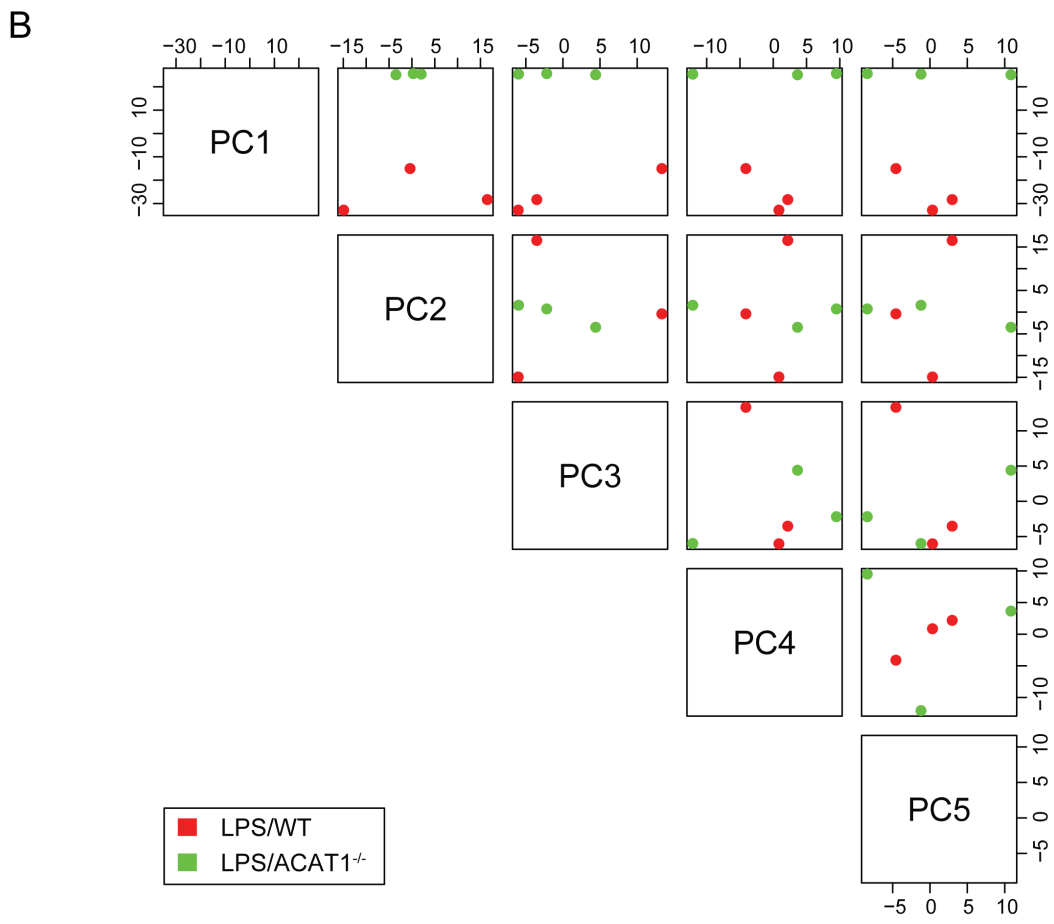

Fig. S2. PCA plots of the differentially expressed (DE) genes comparing WT *vs* ACAT1$^{-/-}$ cells.

Unsupervised principal component analysis (PCA) performed on DE genes ($p<0.05$) for indicated comparisons (n=3 mice per group). (A) PCA plots of DE genes from WT *vs* ACAT1$^{-/-}$ under basal state (no stimulation). (B) PCA plots of DE genes from WT *vs* ACAT1$^{-/-}$ following LPS stimulation (100ng/mL, 2hr).

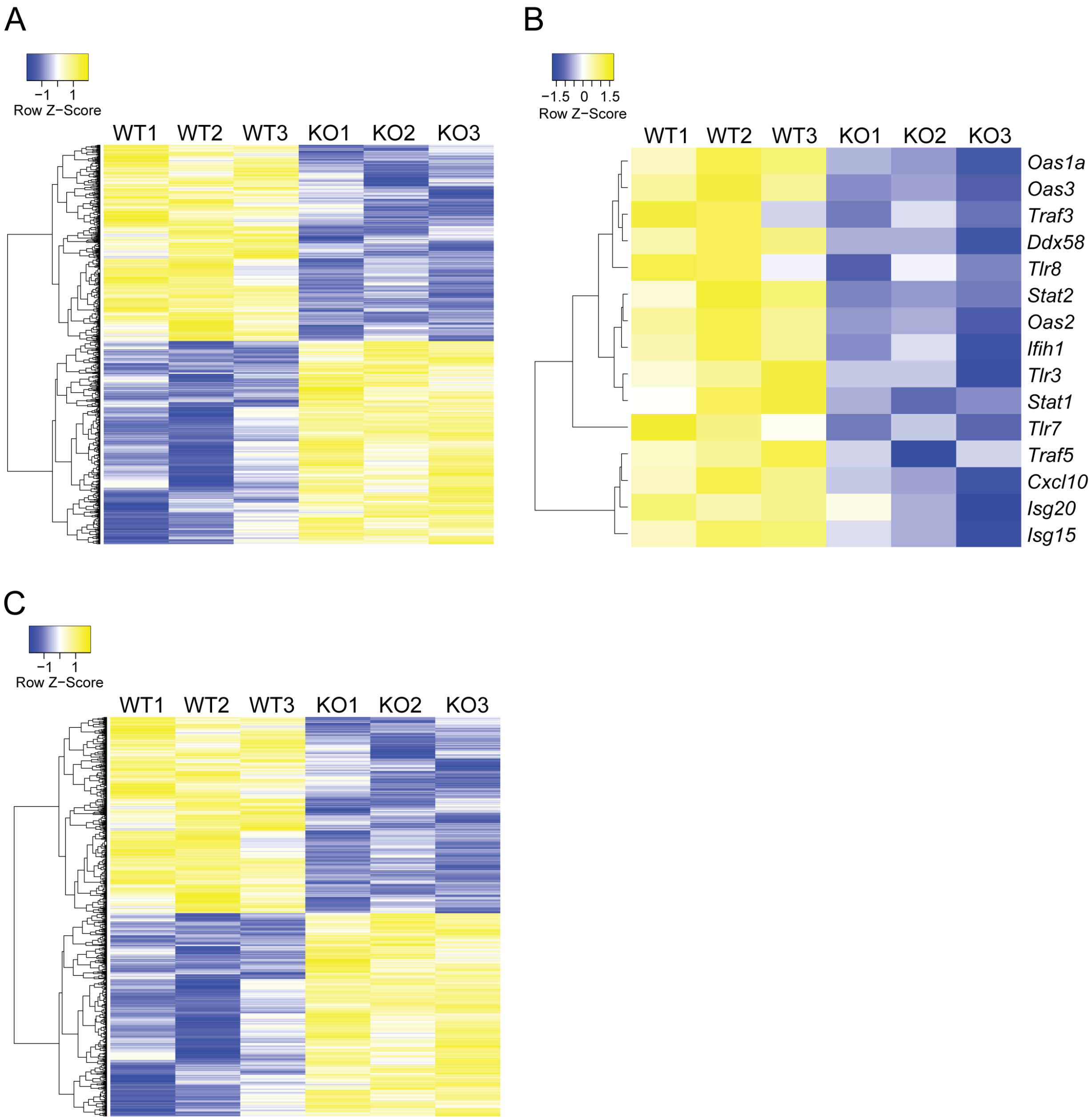

Fig. S3. Heatmaps on the differentially expressed (DE) genes comparing WT *vs* ACAT1-/- cells.

(A) Heatmap of DE genes comparing unstimulated WT *vs* ACAT1$^{-/-}$ cells. (B) Heatmap of the type 1 interferon pathway-related DE genes comparing unstimulated WT *vs* ACAT1$^{-/-}$ cells. (C) Heatmap of DE genes comparing LPS-stimulated WT *vs* ACAT1$^{-/-}$ cells.

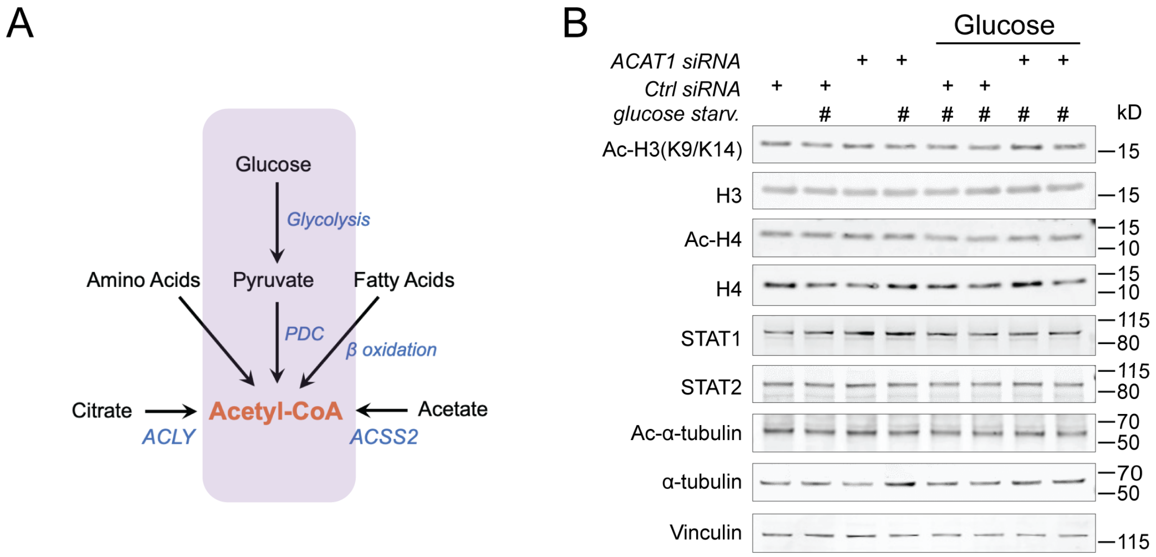

Fig. S4. Glucose mediated histone acetylation is intact in ACAT1 deficient cells.
(A) Scheme summarizing the major metabolic pathways to generate acetyl-CoA with glucose oxidation pathway highlighted in light purple. (B) Western blotting of histone acetylation in human monocytes transfected with either control siRNA or ACAT1 siRNA under glucose starvation in combination with glucose (11.11mM).

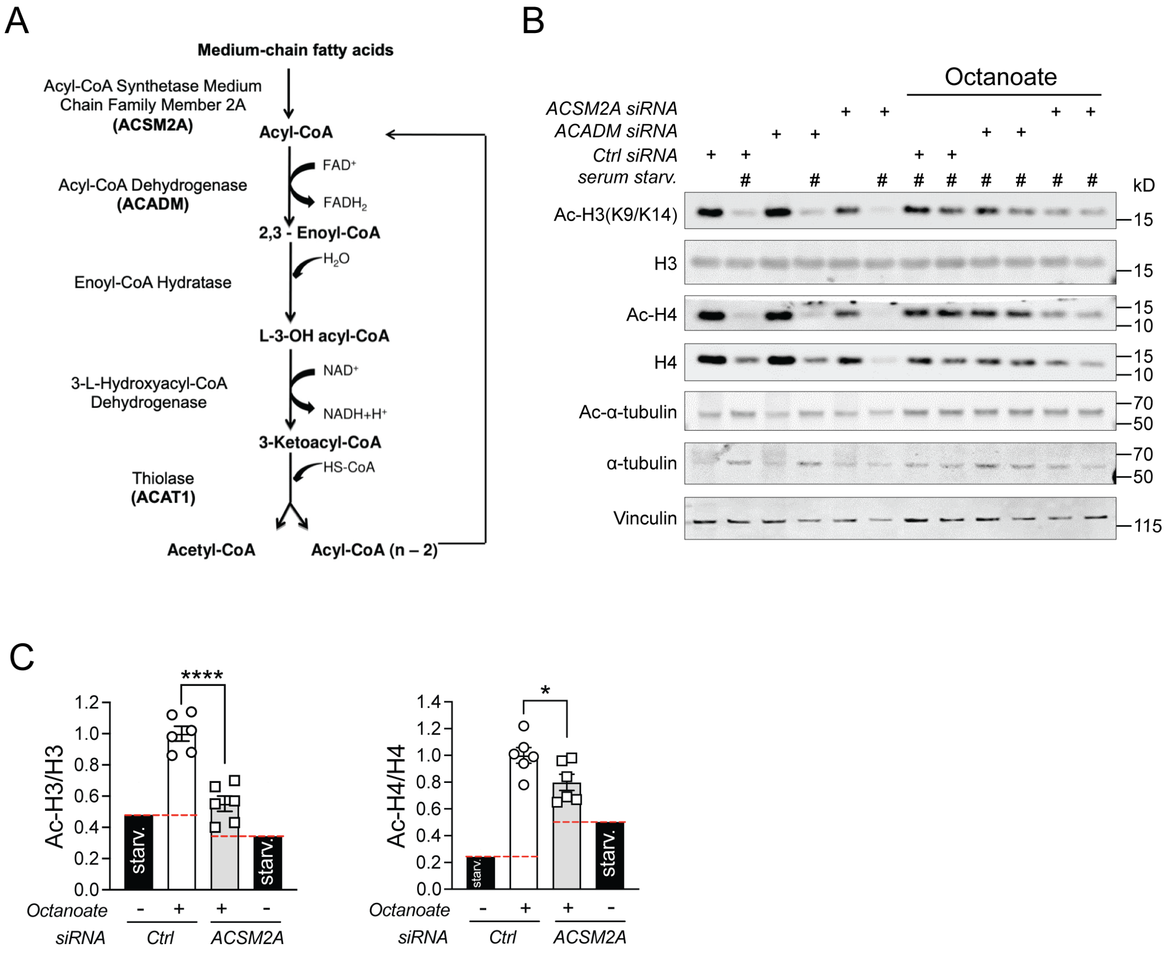

Fig. S5. Fatty acids mediated histone acetylation is impaired in ACSM2A deficient cells. (A) Diagram showing medium-chain fatty acids β-oxidation pathway and key enzymes. (B) Western blotting of histone acetylation in human monocytes transfected with either control siRNA, *ACADM* siRNA or *ACSM2A* siRNA under serum starvation in combination with octanoate (2mM). (C) Quantitative analysis of acetylated H3 to total H3 or acetylated H4 to total H4 ratio in control siRNA or *ACSM2A* siRNA transfected cells. Data were analyzed by unpaired two-tailed Student's t-test. All data were represented as mean ± SEM. *$p<0.05$; ****$p<0.0001$.

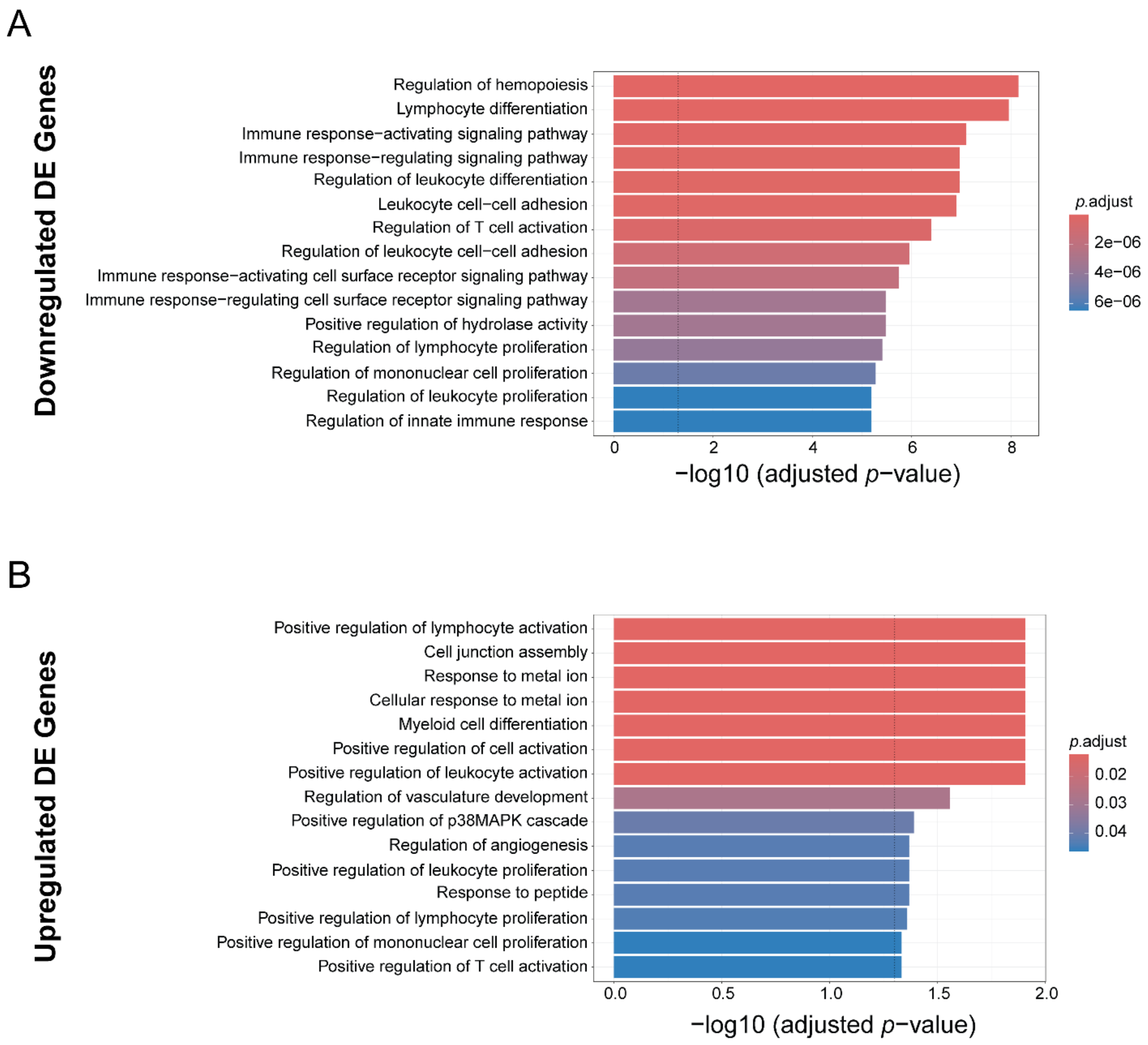

Fig. S6. ATAC-seq pathway enrichment in LPS-stimulated WT and ACAT1-/- cells.
(A) Bar plot of cluster profiler pathways from significantly downregulated DE genes comparing LPS-stimulated WT *vs* ACAT1-/- cells. (B) Bar plot of cluster profiler pathways from significantly upregulated DE genes comparing LPS-stimulated WT *vs* ACAT1$^{-/-}$ cells. The x axis represents negative log10-transformed adjusted *p* values, and the bar-plot color was scaled to transformed adjusted *p* values.

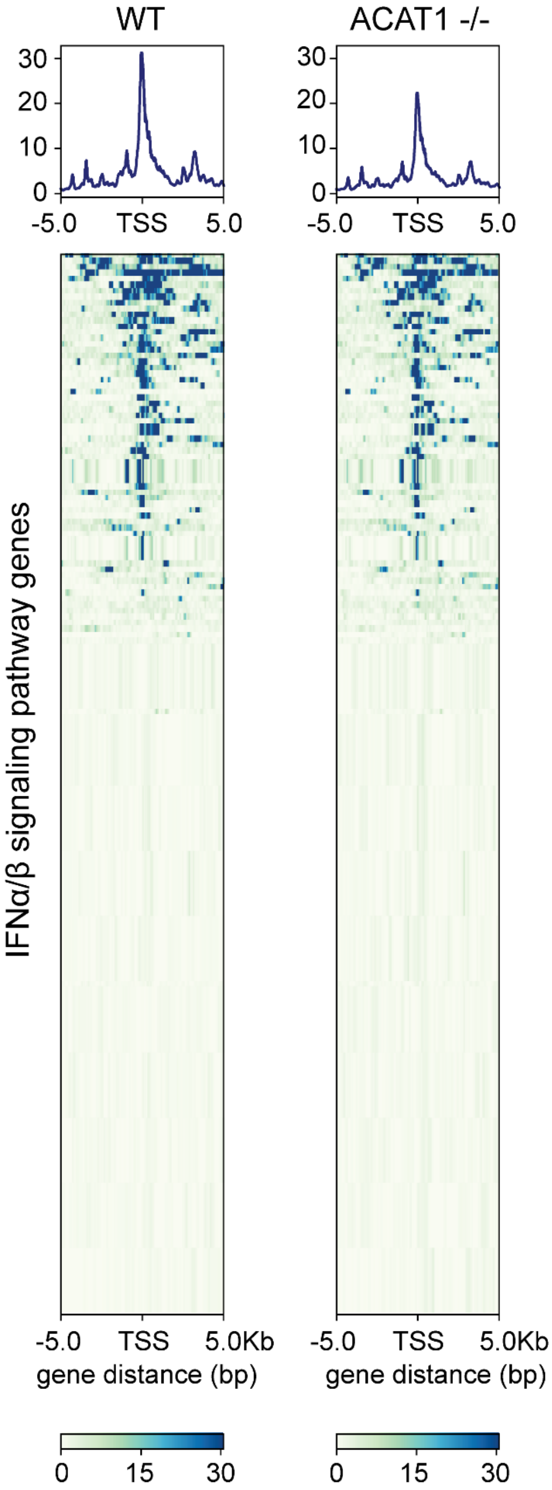
WT
ACAT1 -/-
30
20
10
0
-5.0
TSS
5.0
IFNα/β signaling pathway genes
-5.0
TSS
5.0Kb
gene distance (bp)
0
15
30

Fig. S7. Heatmap and normalized signal density in aligned TSS and flanking regions of IFNα/β signaling pathway genes in WT *vs* ACAT1$^{-/-}$ cells.

Normalized coverage centered around the TSS and the 5kb flanking regions for genes in the IFNα/β signaling pathway (Reactome: R-MMU-909733). The 1-dimensional signal at the top of the figure is column summation of the bottom plot. The value in the top plots is reads per genomic content (RPGC). The bottom plots are colorized matrix of IFNα/β pathway genes in WT and ACAT1$^{-/-}$ cells with LPS stimulation. Average density signal at the TSS in WT cells is of a much higher magnitude than that of the KO cells, indicating diminished chromatin accessibility in the KO cells for IFN α/β signaling pathway genes.

Color	UCSC label	ENCODE classification	ENCODE label
red	prom	promoter-like signature	PLS
orange	enhP	proximal enhancer-like signature	pELS
yellow	enhD	distal enhancer-like signature	dELS
pink	K4m3	DNase-H3K4me3	DNase-H3K4me3
blue	CTCF	CTCF-only	CTCF-only

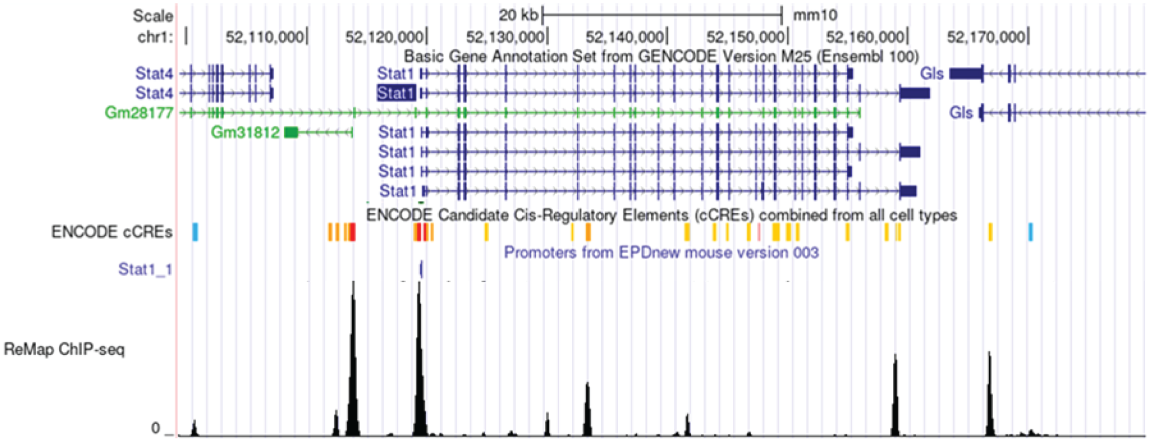

Fig. S8. UCSC Genome Browser on mouse *Stat1* locus.
ENCODE candidate cis-regulatory elements (cCREs) are colorized and indicated at the mouse *Stat1* genomic locus. Various signal peaks identified by CHIP-seq overlap with the ENCODE cCREs sites, including promoter-like signature (PLS), proximal enhancer-like signature (pELS), and distal enhancer-like signature (dELS).

A

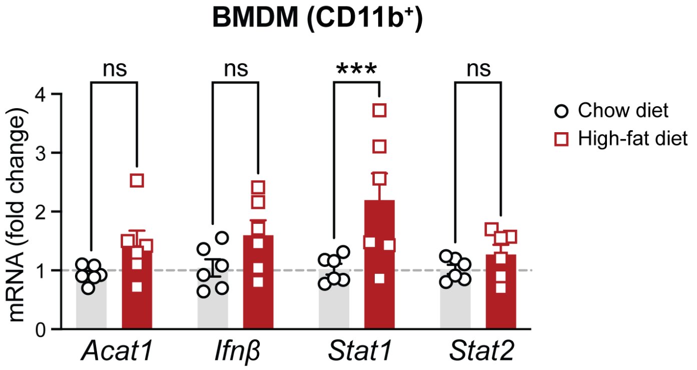

B

Subjects	ID	Age	BMI (kg/m²)
Lean	C01	33	19.7
	C02	23	24.6
	C03	31	20.8
	C07	59	24.4
	C08	59	22.1
Obese	Ob02	60	30.0
	Ob03	53	31.8
	Ob04	48	31.2
	Ob05	57	31.8
	Ob06	51	36.0
	Ob07	41	31.9
	Ob09	35	31.1

Fig. S9. *Stat1* is elevated in $CD11b^+$ BMDM derived from high-fat diet (HFD)-fed mice and lean/obese subjects enrolled in the study.
(A) Quantitative RT-PCR analysis of *Acat1* and type 1 interferon pathway related genes in $CD11b^+$ macrophages isolated from mouse bone marrow (BMDM) fed with chow diet or high-fat diet (HFD) (n=6 mice/group). Data were normalized to 18S rRNA and represented as mean ± SEM. Two-way ANOVA followed by Tukey's multiple comparisons test. ***p<0.001. (B) Age and BMI of Lean or Obese subjects (male, n=5-7 per group) enrolled in this study.

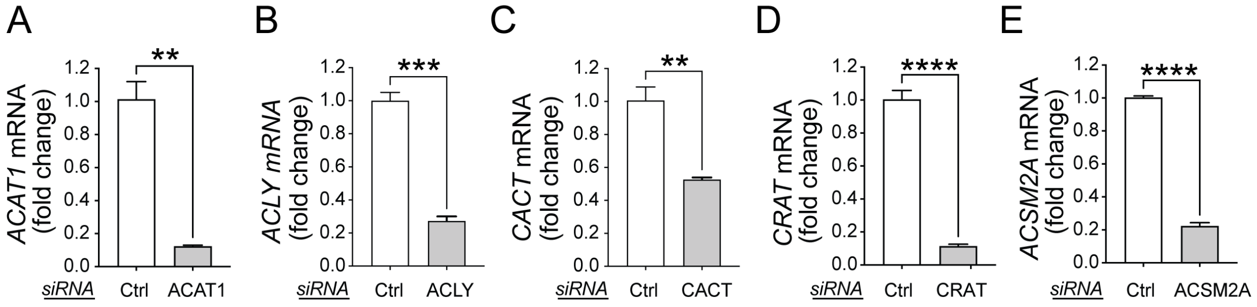

Fig. S10. Validation of various genes' siRNA knockdown by qRT-PCR.
mRNA levels of *ACAT1* (A), *ACLY* (B), *CACT* (C), *CRAT* (D) and *ACSM2A* (E) were reduced by over 50% in human monocytes with corresponding siRNA nucleofection as compared to control siRNA nucleofection. Data were analyzed by unpaired two-tailed Student's *t*-test. All data were represented as mean ± SEM. **p<0.01; ***p<0.001; ****$p<0.0001$.

Table S1. Differentially expressed genes comparing WT *vs* $ACAT1^{-/-}$ under basal state.

Table S1 is provided as a separate file (Table S1.xlsx).

Table S2. Differentially expressed genes comparing WT *vs* $ACAT1^{-/-}$ upon LPS stimulation.

Table S2 is provided as a separate file (Table S2.xlsx)

Table S3. Normalized Precursor M+0 Intensities of the Single Acetylated Peptides (mean± SD).

	K9-Ac-K14-Me3	K9-Me-K14-Ac	K9-Me2-K14-Ac	K9-Ac-K14-Me2	K9-Pr-K14-Ac
WT_Untreated	1.02E+10 ± 8.94E+08	1.79E+10 ± 1.43E+09	5.14E+10 ± 4.39E+09	5.1E+10 ± 2.75E+09	3.08E+10 ± 1.03E+09
WT_Octanoate	6.84E+09 ± 1.72E+08	1.02E+10 ± 8.62E+08	3.15E+10 ± 1.31E+09	3.21E+10 ± 1.22E+09	1.97E+10 ± 1.48E+09
ACAT1$^{-/-}$_Untreated	7.66E+09 ± 8.00E+08	1.7E+10 ± 2.29E+09	4.01E+10 ± 5.57E+09	4.1E+10 ± 5.84E+09	2.94E+10 ± 4.06E+09
ACAT1$^{-/-}$_Octanoate	5.92E+09 ± 5.02E+08	1.27E+10 ± 1.77E+09	3.05E+10 ±1.95E+09	3.11E+10 ± 2.05E+09	2.19E+10 ± 3.84E+09

Table S4. Normalized ^{13}C-Acetyl Precursor Intensities of the Single Acetylated Peptides (mean± SD).

	K9-Ac-K14-Me3	K9-Me-K14-Ac	K9-Me2-K14-Ac	K9-Ac-K14-Me2	K9-Pr-K14-Ac
WT_Untreated	2.89E+09 ± 2.24E+08	5.00E+09 ± 3.54E+08	1.37E+10 ± 1.16E+09	1.36E+10 ± 7.62E+08	8.51E+09 ± 2.81E+08
WT_Octanoate	5.32E+09 ± 3.25E+08	8.07E+09 ± 7.99E+08	2.46E+10 ± 7.65E+08	2.51E+10 ± 6.26E+08	1.52E+10 ± 1.25E+09
ACAT1$^{-/-}$_Untreated	2.32E+09 ± 9.49E+07	4.85E+09 ± 5.57E+08	1.08E+10 ± 1.47E+09	1.11E+10 ± 1.51E+09	8.19E+09 ± 1.01E+09
ACAT1$^{-/-}$_Octanoate	3.55E+09 ± 1.57E+08	6.94E+09 ± 8.61E+08	1.81E+10 ± 2.47E+08	1.81E+10 ± 3.21E+08	1.14E+10 ± 1.69E+09

Table S5. Western Blotting Antibodies Used in this study.

ANTIBODIES	SOURCE	IDENTIFIER
Rabbit monoclonal anti-IRF3[EPR2418Y]	Abcam	Cat# ab68481; RRID:AB_11155653
Rabbit monoclonal anti-STAT1	Cell Signaling	Cat# 9172, RRID:AB_2198300
Rabbit monoclonal anti-STAT1(phosphoY701) (58D6)	Cell Signaling	Cat# 9167, RRID:AB_561284
Rabbit monoclonal anti-STAT2 (D9J7L)	Cell Signaling	Cat# 72604, RRID:AB_2799824
Rabbit polyclonal anti-STAT2 (phosphoY689)	Millipore Sigma	Cat# 07-224, RRID: AB_2198439
Rabbit polyclonal anti-Acetyl-Histone H3 (Lys9/Lys14)	Cell Signaling	Cat# 9677, RRID: AB_1147653
Rabbit polyclonal anti-Histone H3	Cell Signaling	Cat# 9715, RRID: AB_331563
Rabbit polyclonal anti-Acetyl-Histone H4 (Lys5/Lys8/Lys12/Lys16)	Millipore Sigma	Cat# 06-866, RRID: AB_310270
Mouse monoclonal anti-Histone H4 (L64C1)	Cell Signaling	Cat#: 2935, RRID: AB_1147658
Goat polyclonal anti-ACAT1	Thermo Fisher Scientific	Cat#: PA5-19227, RRID: AB_10977900
Mouse monoclonal anti-Vinculin	Sigma-Aldrich	Cat#: V4505, RRID: AB_477617
Rabbit monoclonal anti-Acetyl-α-Tubulin (Lys40) (D20G3) XP®	Cell Signaling	Cat# 5335, RRID: AB_10544694
Mouse monoclonal anti-α-Tubulin (DM1A)	Cell Signaling	Cat# 3873, RRID: AB_1904178
